# Adaptive and Innate Immunity Are Key Drivers of Age at Onset of Multiple Sclerosis

**DOI:** 10.1212/NXG.0000000000200159

**Published:** 2024-05-29

**Authors:** Elina Misicka, Yunfeng Huang, Stephanie Loomis, Nilanjana Sadhu, Elizabeth Fisher, Arie Gafson, Heiko Runz, Ellen Tsai, Xiaoming Jia, Ann Herman, Paola G. Bronson, Tushar Bhangale, Farren B. Briggs

**Affiliations:** From the Department of Population and Quantitative Health Sciences (E.M.), Case Western Reserve University, Cleveland, OH; Biogen (Y.H., S.L., N.S., E.F., A.G., H.R., E.T., P.G.B.), Cambridge, MA; Human Genetics and Bioinformatics (X.J., A.H., T.B.), Genentech, San Francisco, CA; and Department of Public Health Sciences (F.B.B.), University of Miami, FL.

## Abstract

**Background and Objectives:**

Multiple sclerosis (MS) age at onset (AAO) is a clinical predictor of long-term disease outcomes, independent of disease duration. Little is known about the genetic and biological mechanisms underlying age of first symptoms. We conducted a genome-wide association study (GWAS) to investigate associations between individual genetic variation and the MS AAO phenotype.

**Methods:**

The study population was comprised participants with MS in 6 clinical trials: ADVANCE (N = 655; relapsing-remitting [RR] MS), ASCEND (N = 555; secondary-progressive [SP] MS), DECIDE (N = 1,017; RRMS), OPERA1 (N = 581; RRMS), OPERA2 (N = 577; RRMS), and ORATORIO (N = 529; primary-progressive [PP] MS). Altogether, 3,905 persons with MS of European ancestry were analyzed. GWAS were conducted for MS AAO in each trial using linear additive models controlling for sex and 10 principal components. Resultant summary statistics across the 6 trials were then meta-analyzed, for a total of 8.3 × 10^−6^ single nucleotide polymorphisms (SNPs) across all trials after quality control and filtering for heterogeneity. Gene-based tests of associations, pathway enrichment analyses, and Mendelian randomization analyses for select exposures were also performed.

**Results:**

Four lead SNPs within 2 loci were identified (*p* < 5 × 10^−8^), including a) 3 SNPs in the major histocompatibility complex and their effects were independent of HLA-DRB1*15:01 and b) a *LOC105375167* variant on chromosome 7. At the gene level, the top association was *HLA-C* (*p* = 1.2 × 10^−7^), which plays an important role in antiviral immunity. Functional annotation revealed the enrichment of pathways related to T-cell receptor signaling, autoimmunity, and the complement cascade. Mendelian randomization analyses suggested a link between both earlier age at puberty and shorter telomere length and earlier AAO, while there was no evidence for a role for either body mass index or vitamin D levels.

**Discussion:**

Two genetic loci associated with MS AAO were identified, and functional annotation demonstrated an enrichment of genes involved in adaptive and complement immunity. There was also evidence supporting a link with age at puberty and telomere length. The findings suggest that AAO in MS is multifactorial, and the factors driving onset of symptoms overlap with those influencing MS risk.

## Introduction

The first symptoms of multiple sclerosis (MS) typically present between the ages of 20 and 40 years, although age at onset (AAO) is highly variable. MS AAO is a significant predictor of several clinical outcomes, including disease activity, time to disability milestones, time to transition from relapsing to progressive disease, and disease severity.^1-4^ Epidemiologic studies suggest that birth in winter and in low radiation geographies, low sunlight exposure, lower education, and high body mass index (BMI) may contribute to earlier AAO.^5-7^ As of yet, the biological mechanisms underlying MS onset remain unclear, as are the mechanisms that connect AAO with subsequent outcomes. Thus, investigating AAO may advance our understanding of the early mechanisms at play in MS and may provide novel insights into potential drivers of disease severity and progression.

AAO of MS is likely multifactorial, with a prominent genetic component. AAO is strongly correlated in monozygotic twins (*r* = 0.6), and the correlations among relatives are proportional to their degree of relatedness.^8^ Familial cases have been shown to present with symptoms earlier than sporadic cases,^9^ suggesting the genetic mechanisms underlying risk may contribute to shortening the subclinical phase of MS and/or predisposing individual for an earlier AAO. This is supported by studies reporting an association between earlier MS onset and both HLA-DRB1*15:01, the primary MS risk allele, and a higher genetic risk score of ∼200 nonmajor histocompatibility complex (MHC) MS risk variants.^10-12^ There have been 2 large, published genome-wide association studies (GWAS) for MS AAO. The earlier study was incorporated into the later analysis of ∼465,000 single nucleotide polymorphisms (SNPs) in 9,772 persons with MS (PwMS) of European ancestry.^13,14^ One variant met genome-wide significance and was in high linkage disequilibrium (LD; EUR *r*^*2*^ = 0.70) with HLA-DRB1*15:01; no other locus contained SNPs with *p*-values <1 × 10^−5 14^. Here, we present an independent GWAS for AAO leveraging 6 well-defined clinical trials consisting of 3,905 PwMS of European ancestry and spanning 8.3 million SNPs, an 18-fold increased coverage from the prior GWAS. We also incorporated gene-based tests of association, pathway enrichment analyses, and Mendelian randomization analyses to examine the relationships between MS AAO and other selected exposures (including known MS risk factors).

## Methods

### Study Population and Genotyping

The study population consisted of 3,905 PwMS from 6 clinical trials: the ADVANCE, ASCEND, and DECIDE studies by Biogen and the OPERA1**,** OPERA2, and ORATORIO studies by Genentech (Table 1; eTables 1–2A).^15^ AAO was generated by subtracting years since onset of symptoms from age at the enrollment in ADVANCE and by subtracting the birth year from year of first observed symptom in ASCEND and DECIDE, collected by trained study personnel. AAO for the OPERA1, OPERA2, and ORATORIO cohorts was also calculated by subtracting birth year from year of first observed symptom. AAO was rank-normalized (eFigure 1).

**Table 1:** Study Population and Study Cohort Characteristics

Trait	Biogen clinical trials			Genentech clinical trials			
	ADVANCE	ASCEND	DECIDE	OPERA1	OPERA2	ORATORIO	Overall
Sample size (N)	690	582	1,076	528	529	500	3,905
Onset age (y; mean, [SD])	31 (8.5)	32 (8.2)	31 (8.7)	31 (9.3)	31 (9.2)	39 (8.1)	31 (9.2)
Genotyping platform	Affymetrix UK Biobank Axiom Array			Illumina HiSeq, 30x			n/a
Imputation approach	Michigan Imputation Server and 1000 G phase 3v5			n/a			n/a
SNPs (N)	9.7×10^6^	9.4×10^6^	9.4×10^6^	8.6×10^6^	8.6×10^6^	8.5×10^6^	8.3×10^6^
MS subtype	RRMS	SPMS	RRMS	RRMS	RRMS	PPMS	

DNA samples for participants from each trial were extracted from blood. The Affymetrix UK Biobank Axiom Array was used to separately genotype the ADVANCE and DECIDE cohorts and the ASCEND cohort (eMethods). Imputation was performed for each batch on the University of Michigan imputation server. Principal components analysis and identification of genetic outliers was performed using Eigensoft v7.2.1. Whole genome sequencing of OPERA1, OPERA2, and ORATORIO (mean read depth of 30×) was performed with Illumina HiSeq. Principal components analysis was conducted using Eigensoft v7.2.1. Resulting alignments were analyzed using GATK^16^ for base quality score recalibration, realignment of indels, removal of duplicates, and SNP/INDEL discovery. See eMethods for exclusion criteria for SNPs and indels for all cohorts. In total, 8,305,328 SNPs were analyzed.

### Genome-Wide Association and Meta-Analyses

GWAS were conducted separately in each trial where rank-normalized AAO was the dependent variable in a linear regression model where the independent variable of interest was an additively coded genotype (0,1,2), and other covariates were sex and the top 10 eigenvectors from PCA using PLINK v1.9.^17^ SNPs were excluded if they had a minor allele frequency ≤0.01, Hardy-Weinberg equilibrium *p*-value<1.0 × 10^−50^, or an imputation INFO score ≤0.30. Summary statistics for the effect size and standard errors for the genotype term were calculated in each trial and used in a fixed-effects meta-analysis model implemented in PLINK v1.9. We retained results for 8,292,283 SNP associations with I^2^ heterogeneity values < 75%. LocusZoom was used visualize regional information for top associations.^18^ FIVEx, LDlink, Human Protein Atlas, and NHGRI-EBI GWAS Catalog were evaluated to guide interpretations.^19-22^ We examined whether the effects for our top hits were independent of HLA-DRB1*15:01 or whether they varied across number of HLA-DRB1*15:01 alleles (i.e., if there was an interaction). In addition, we extracted the associations for the top AAO hits from the most recent summary statistics for MS risk, severity (age-related MS severity score), and longitudinal brain volume and T2 lesion volume.^4,15,23^ We similarly extracted the genome-wide significant or top-ranking SNPs from the GWAS for MS risk, severity, and changes in brain/lesion volume and report their associations with AAO. If SNPs of interest were not available in the respective summary statistics, suitable proxies were identified based on LD (1000 Genomes EUR hg38).

### Gene-Based Tests of Association

Gene-based tests of association were performed using MAGMA implemented in FUMA,^24,25^ implementing gene boundaries of ±10 kilobases (kb). MAGMA creates a gene-based test statistic by converting *p*-values of SNP-level summary statistics to χ^2^ values, which are averaged across SNPs mapped to a gene range. The mean χ^2^ is then converted to a *p*-value to determine a gene-based level of association.

#### Pathway Enrichment Analyses

Pathway enrichment analyses were completed for 3 gene sets. The first gene set (GS1) consisted of unique genes in which genic SNPs (as defined by MAGMA) had an AAO association *p*-value <0.0001; genic SNPs were defined using exact gene boundaries (no flanking regions). SNP annotation was performed using SeattleSeq.^26^ The second gene set (GS2) consisted of genes associated with AAO with *p*-value <0.01 from the gene-based association tests. Finally, the union of GS1 and GS2 comprised the third gene set (GS3). Pathway enrichment analysis was performed in Enrichr,^27^ using the REACTOME 2022,^28^ the KEGG 2021 Human,^29^ and the Human Molecular Signatures Hallmark 2020^30^ databases to characterize pathways and biological relationships enriched in GS1 through GS3. Enrichr creates an odds ratio for each enriched pathway from the z-score of the SD of the expected rank, as calculated by a Fisher exact test for many random gene sets in the gene-set library. The natural logarithm of the *p*-value from the Fisher exact test is then multiplied by the z-score to calculate the “Combined Score”. A multiple testing adjusted *p*-value (Benjamini-Hochberg) was used to guide interpretations.

### Mendelian Randomization

Two-sample Mendelian randomization (MR) was conducted to investigate the effects of select MS risk factors on MS AAO using an inverse variance-weighted–based approach. Specifically, the directional effects of BMI,^31^ serum vitamin D levels,^32^ age at puberty,^33^ and telomere length^34^ were explored as variation within these traits have been associated with MS risk.^35-39^ Analysis was performed using the R package *TwoSampleMR v0.5.6*.^40^ Genetic instruments were selected from publicly available summary statistic data sets from GWAS in populations with European ancestry (eTable 2B). The effects of horizontal pleiotropy were investigated using the MR-Egger intercept test.

### Standard Protocol Approvals, Registrations, and Patient Consents

We performed a GWAS meta-analysis of AAO among 3,905 PwMS who were participants in the 6 clinical trials listed in the methods section. All participants provided written informed consent that covered the scope of this research. Ethical approval was provided as per the original RCTs. STREGA reporting guidelines were implemented during the completion of this manuscript.^41^

### Data Availability

Genome-wide summary statistics are available on request. Requests may be sent to the corresponding author and Yunfeng Huang, yunfeng.huang@biogen.com.

## Results

### Meta-Analysis of Genome-Wide Association Summary Statistics

**Figure 1:**
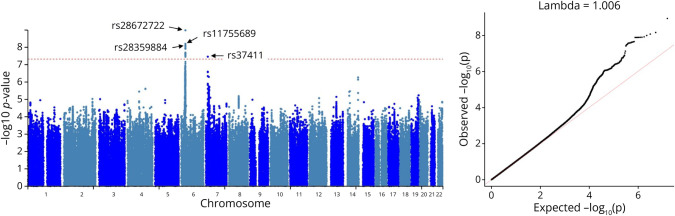
Manhattan Plot and Normal-QQ Plot for MS Age at Onset SNP Associations

In our meta-analysis of 8.3 million SNPs, 26 associations across 2 loci met genome-wide significance (eTable 3) and included 4 independent signals (*r*^*2*^ < 0.3) with consistent direction of effects across the 6 trials (*p* < 5.0 × 10^−8^; Table 2, Figure 1, eFigures 2–5, eTable 3). The first locus spanned 43.5 kb within the MHC and included 3 independent signals (*r*^*2*^ < 0.3), which were not in LD with HLA-DRB1*15:01 (*r*^*2*^ < 0.08), the top MS susceptibility variant (eTable 4A). The associations for these 4 independent signals persisted (though the 3 MHC signals slightly attenuated [<15%]) when adjusting for HLA-DRB1*15:01, and there was no evidence that their effects varied according to the number of HLA-DRB1*15:01 risk alleles (eTable 4B).

**Table 2:** Genomic Risk Loci and Independent Significant SNPs From the Meta-Analysis of GWAS Summary Statistics From 6 Cohorts

Chr	Base pair position (genome build hg38)	rsID	MAF^a^	Effect allele^b^	Other allele	Beta	SE	*p* value	Q	*I* ^2^	Gene	SNP function
6	32658660	rs28672722	0.215	T	G	0.1657	0.0272	1.11 × 10^−9^	0.38	5.96	*HLA-DQB1*	<1 kb downstream
6	32618699	rs11755689	0.294	G	A	0.142	0.0245	6.65 × 10^−9^	0.88	0.00	*HLA-DQA1*	<10 kb upstream
6	32616653	rs28359884	0.432	A	C	0.135	0.0234	8.20 × 10^−9^	0.84	0.00	*-*	Intergenic
7	15740071	rs37411	0.066	T	C	−0.2528	0.0459	3.64 × 10^−8^	0.21	29.59	*HSALNG0056337* (lncRNA)	Intron variant

a MAF: minor allele frequency.

b Effect allele is also the minor allele.

Rs28672722T (<1 kb downstream *HLA-DQB1*; 1 kb downstream *HLA-DQA1*) was associated with a later AAO of MS (β = 0.166, *p* = 1.11 × 10^−9^), as were rs11755689 G (9 kb upstream *HLA-DQA1*) and rs28359884A (21 kb upstream *HLA-DQA1*) (Table 2). These variants were expression quantitative trait loci (eQTLs) for several MHC Class II genes across multiple tissues (*p* < 5.0 × 10^−8^). SNPs rs28672722 and rs11755689 were associated with increased expression of *HLA-DQA1* and *HLA-DQB1* and decreased expression of *HLA-DQA2* and *HLA-DQB2*, while rs11755689 was associated with decreased *HLA-DRB5* expression but rs28672722 was also associated with increased expression of *HLA-DRB5* and *HLA-DRB1* and decreased expression of *TAP2* and *HLA-DOB* (eTables 5A and 6). Of interest, rs28359884 had nearly reversed relationships as an eQTL because it was associated with decreased expression of *HLA-DQA1* and *HLA-DRB1* and increased expression of *HLA-DQA2* (eTable 7A). SNPs rs28672722 and rs28359884 were also in perfect LD (*r*^*2*^ = 1) with eQTLs for these HLA genes, while rs285359884 was in LD with eQTLs for *PRRT1*, *LY6G5B*, *HLA-DRB6*, *CYP21A1P*, and *C4A* (*p* < 5.0 × 10^−8^; eTables 5B and 7B).

The second locus was on chromosome 7p21.2, with rs37411T, a long noncoding RNA (lncRNA *HSALNG0056337*; *ENSG00000286376*) intronic variant, associated with an earlier AAO of MS (β = −0.253, *p* = 3.64 × 10^−8^). This variant is negatively associated with expression of *MEOX2* and positively with *AGMO* expression (*p* < 0.05; eTable 8). Other non-MHC loci with suggestive independent associations (*p* < 1.0 × 10^−6^; eTable 3) include an intergenic variant on chromosome 7p15.3, rs6461685T, that is associated with the expression of adjacent genes: *KLHL7*, *KLHL7-DT*, *NUPL2*, and *GPNMB*, and rs144445292A and intronic *HHIPL1* variant on 14q32.2 (data not shown).

For the 3 MHC SNPs, the major alleles (rs28672722 G, rs11755689A, rs28359884C) were associated with earlier AAO, while it was the minor allele for non-MHC SNP (rs37411T) (Table 2). In examination of the MS risk summary statistics, the major alleles associated with earlier AAO were also associated with an increased risk for MS (eTable 9A). In examination of the MS severity summary statistics, proxies for rs28359884C (*r*^*2*^ = 1) were associated with greater MS severity and would survive a false discovery rate correction (*p* < 0.0125; eTable 9B). None of our top AAO hits were associated with longitudinal changes in brain volume or T2 lesion volume (eTable 9C).

Of the 232 established risk variants for MS, 213 SNPs were available or had LD proxies in the MS AAO data set (eTable 9D). The most significant association was rs3135388A, which tags HLA-DRB1*15:01 and was associated with earlier AAO (*p* = 5.8 × 10^−4^), consistent with the prior literature. Of the other risk variants, 7% of them had marginal associations with AAO (*p* < 0.05), but none would survive multiple testing correction. The tops hits from the MS severity GWAS and the MRI study were not associated with AAO (eTables 9, E and F).

### Gene-Based Test Results

**Figure 2:**
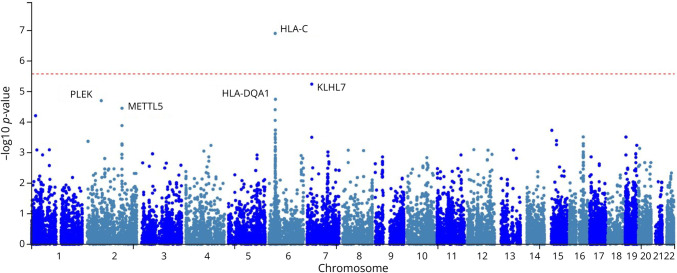
Manhattan Plot for MS Age at Onset Gene-Based Tests of Association

Gene-based tests of association were conducted for >19,000 genes (eTable 10). Top gene-based test results were for *HLA-C* and *HLA-DQA1* in the MHC (*p* = 1.23 × 10^−7^ and *p* = 1.77 × 10^−5^, respectively), with *HLA-C* as the only gene to clear a Bonferroni multiple testing threshold of *p* < 2.6 × 10^−6^) and for non-MHC genes *KLHL7* on 7p15.3 (7:23105785-23177914 [hg38] *p* = 5.64 × 10^−7^), *PLEK* on 2p14 (2:68582305-68634585 [hg38]; *p* = 1.96 × 10^−5^), and *METTL5* on 2q31.1 (2:170656591-17069144 [hg38]; *p* = 3.48 × 10^−5^) (Figure 2). Multiple other HLA and non-HLA genes within the MHC and non-MHC genes implicated by the top SNP associated also had significant gene-based associations with AAO (*p* < 0.05), including *HLA-DRB1*, *HLA-DQB1*, *MEOX2*, *NUPL2*, *GPNMB*, *C4A*, and others, such as *CYP2R1*, a hydroxylase associated with vitamin D metabolism (see eTable 10).

### Pathway Enrichment Analysis Results

GS1, GS2, and GS3 consisted of 157, 319, and 438 genes, respectively (eTable 11). As anticipated, there was an enrichment of pathways involving HLA genes across gene sets (Table 3, eTables 12–14). For example, in the REACTOME and KEGG databases several of the significant pathways (p_Benjamini-Hochberg[BH]_<0.05) for GS1-3 that included HLA genes were related to T-cell receptor signaling, autoimmunity, and interferon (IFN) gamma signaling. There was also an enrichment of complement cascade and other immune signaling pathways across the databases including REACTOME: *Activation of C3 and C5* (GS2 p_BH_ = 0.02), MSigDB: *Complement* (GS2 p_BH_ = 0.05), and MSigDB: *TNFα Signaling* through *NK-κB* (p_BH_ = 0.05). There were several nonimmune response pathways with high combined scores, such as KEGG: *Non-homologous end-joining* (GS1 p_BH_ = 0.04), KEGG: Cortisol synthesis and secretion (GS2 p_BH_ = 0.04), and MSigDB: *Coagulation* (GS2 p_BH_ = 0.0032).

**Table 3:** Pathway Enrichment Analysis Results for GS3

Database	Term	Genes^a^	Overlap^b^	OR^c^	Combined Score^d^	p(GS3)^e^	p(GS1)^e^	p(GS2)^e^
REACTOME	PD-1 Signaling R-HSA-389948	*HLA-DRB5, HLA-DRA, PTPN11, HLA-DQA1, HLA-DRB1*	5/21	14.1	134.0	7.50 × 10^−5^	0.011	1.55 × 10^−5^
	Activation Of C3 And C5 R-HSA-174577	*C4B, C4A, CFB*	3/6	45.0	383.3	1.99 × 10^−4^	—	7.47 × 10^−5^
	Translocation Of ZAP-70 To Immunological Synapse R-HSA-202430	*HLA-DRB5, HLA-DRA, HLA-DQA1, HLA-DRB1*	4/17	13.9	107.4	4.31 × 10^−4^	0.008	1.22 × 10^−4^
	Interferon Gamma Signaling R-HSA-877300	*SP100, HLA-DRB5, HLA-B, HLA-C, HLA-DRA, PTPN11, HLA-DQA1, HLA-DRB1*	8/89	4.5	32.2	7.45 × 10^−4^	0.033	8.09 × 10^−5^
	Phosphorylation Of CD3 And TCR Zeta Chains R-HSA-202427	*HLA-DRB5, HLA-DRA, HLA-DQA1, HLA-DRB1*	4/20	11.3	79.8	8.32 × 10^−4^	0.011	2.40 × 10^−4^
KEGG	Type I diabetes mellitus	*IL1A, HLA-DRB5, PTPRN2, HLA-B, HLA-C, HLA-DRA, HLA-DQA1, HLA-DRB1, INS, HLA-DQB1*	10/43	13.8	243.2	2.30 × 10^−8^	2.12 × 10^−5^	9.86 × 10^−10^
	Graft-versus-host disease	*IL1A, HLA-DRB5, HLA-B, HLA-C, HLA-DRA, HLA-DQA1, HLA-DRB1, HLA-DQB1*	8/42	10.7	135.7	3.05 × 10^−6^	3.24 × 10^−4^	2.57 × 10^−7^
	Allograft rejection	*HLA-DRB5, HLA-B, HLA-C, HLA-DRA, HLA-DQA1, HLA-DRB1, HLA-DQB1*	7/38	10.2	112.9	1.62 × 10^−5^	2.19 × 10^−4^	1.87 × 10^−6^
	Autoimmune thyroid disease	*HLA-DRB5, HLA-B, HLA-C, HLA-DRA, HLA-DQA1, HLA-DRB1, HLA-DQB1*	7/53	6.9	60.7	1.49 × 10^−4^	7.93 × 10^−4^	1.86 × 10^−5^
	Staphylococcus aureus infection	*C4B, C4A, HLA-DRB5, HLA-DRA, CFB, HLA-DQA1, HLA-DRB1, HLA-DQB1, C2*	9/95	4.8	39.6	2.38 × 10^−4^	0.039	1.91 × 10^−5^
MSigDB	Coagulation	*PROC, SERPINB2, DCT, MMP15, TMPRSS6, PLEK, OLR1, MMP8, CFB, SH2B2, C2*	11/138	3.9	33.0	2.33 × 10^−4^	0.295	6.80 × 10^−5^
	Complement	*SERPINB2, MMP15, TMPRSS6, PLEK, OLR1, CDH13, MMP8, HPCAL4, CFB, C2*	10/200	2.4	10.3	0.013	—	0.005
	TNFα Signaling via NF-kB	*KLF10, IL1A, SERPINB2, B4GALT1, DRAM1, PLEK, OLR1, HES1, SOD2*	9/200	2.1	7.3	0.033	0.795	0.005
	Interferon Gamma Response	*VAMP8, HLA-B, RBCK1, SOD2, VAMP5, CFB, HLA-DQA1, HLA-DRB1, PSMB10*	9/200	2.1	7.3	0.033	0.795	0.005
	Heme Metabolism	*RANBP10, TOP1, PDZK1IP1, YPEL5, CLCN3, TNS1, OSBP2, ABCG2, TNRC6B*	9/200	2.1	7.3	0.033	0.073	0.209

a Genes: the list of genes submitted to Enrichr which overlapped with the enriched biological pathway.

b Overlap: the fraction of genes submitted to Enricher that overlapped with the enriched biological pathway over the total number of genes involved in the enriched biological pathway.

c Odds ratio: the ratio describing the proportion of genes assigned to a given pathway as compared with the number expected by chance alone.

d Combined score: calculated by multiplying the natural log of the Fisher exact test *p*-value and the z-score for the expected rank.

e P-values of the Fisher exact test for gene set 1 (GS1; genes containing one or genic SNPs as annotated in SeattleSeq), gene set 2 (GS2; genes enriched for MS AAO using MAGMA gene-based tests of association), and gene set 3 (GS3; the union of gene sets 1 and 2).

### Mendelian Randomization

Mendelian randomization was conducted to investigate the effects of BMI, vitamin D levels, telomere length, and age at puberty on MS AAO (eTables 15–18). Only telomere length and age at puberty demonstrated evidence for a causal influence on MS AAO (*p* < 0.05), with increased telomere length conferring a later AAO (β = 0.220, *p* = 0.008) and later age at puberty conferring later AAO (β = 0.087, *p* = 0.029) (eTable 19). Thus, shorter telomere length and earlier age at puberty were associated with earlier MS AAO. Investigation of horizontal pleiotropy was performed using the MR-Egger Intercept Test. No pleiotropic effects were detected (*p* < 0.05 for all tests). Leave-one-out sensitivity analyses did not detect any individual SNPs driving the observed associations.

## Discussion

The underlying association between AAO and the risk, presentation, and progression of MS is not yet understood, necessitating a deeper understanding of the drivers of MS AAO. In this GWAS leveraging genetic data from 6 clinical trials, 2 genomic loci with 4 independent SNP associations (*r*^*2*^ < 0.3; *p* < 5 × 10^−8^) were associated with MS AAO. The first locus was a 43 kb region within the MHC that spanned *HLA-DQA1*, and the 3 associated SNPs were not in LD with HLA-DRB1*15:01. The second locus was a lncRNA intronic variant on chromosome 7p21.2. There was emerging evidence for associations between these variants and MS risk and for one variant and MS severity from prior studies. Gene-based tests of association using SNP-level AAO summary data revealed an enrichment of several MHC and non-MHC genes involved in adaptive and innate immunity and indicated potential roles for the complement system and cell signaling. There was also evidence from MR analyses that an earlier age of puberty and shorter telomere length conferred an earlier MS AAO. In brief, we identified novel genetic associations with MS AAO that implicate several biological processes and demonstrate the need for additional research to resolve the underlying relationships and the mechanisms that may overlap between MS risk, symptom initiation, disease severity, and progression.

Beyond the established inverse relationship between HLA-DRB1*15:01 and AAO, there was little evidence to suggest a prominent role for other individual risk variants or variants recently associated with MS severity and longitudinal changes in brain volume and T2 lesion load.^4,15,23^ An interesting observation was that the alleles associated with earlier AAO (major alleles for rs28672722, rs11755689, rs28359884; minor allele for rs37411) were also associated with increased risk for MS—this is consistent with our current understanding that an overall higher burden of MS genetic risk variants (whether it be HLA-DRB1*15:01 or a genetic/polygenic risk score) confers an earlier onset of MS.^4,10^ The relationships between these 3 AAO variants and MS risk were not unexpected, considering the complexity of the MHC and that there are >30 independent risk alleles in the region.^23^ Another intriguing observation was that LD proxies for rs28359884C, the major allele associated with earlier onset and increased MS risk, were also associated with higher MS severity. This is consistent with findings published by the International MS Genetics Consortium (IMSGC), where the risk alleles for 2 MHC variants (rs3135388A which tags HLA-DRB1*15:01 and intergenic rs9271366 G that resides between *HLA-DRB1* and *HLA-DQA1* and tags *HLA-DQB1* amino acid substitution^23^) were also associated with higher MS severity measured by age-related MS severity score (*p* < 0.01) and increased hazard ratios for time to reach an Expanded Disability Status Score of 6 (*p* < 0.02)—but these associations did not meet a multiple testing correction in their publication.^4^ These 2 MS risk alleles are not in LD with rs28359884 (*r*^*2*^ < 0.15), and both risk alleles are associated with earlier AAO (eTable 9D), which hints at a possible nuanced MHC connection between MS risk and MS severity via AAO. This later hypothesis is supported, in part, by findings from the IMSGC where polygenic risk scores (combined, MHC alone, non-MHC alone) were positively associated with MS severity, but the relationship substantially attenuated when adjusted for AAO.^4^ Thus, our work highlights the need for additional analyses with greater genetic resolution, particularly in the MHC, to determine whether these observations robust.

The 3 MHC variants associated with AAO are eQTLs for multiple HLA genes, which might highlight one mechanism through which they contribute to both MS risk and AAO. The AAO MHC variant rs28672722 and its neighboring independent signals rs11755689 and rs28359884 are in LD with variants associated with risk for several autoimmune diseases, including Sjogren disease, rheumatoid arthritis, and systemic lupus erythematosus (eTable 20A). In addition, rs11755689 has prior associations with LDL cholesterol and omega-6 polyunsaturated fatty acid, as well as levels of various phospholipids (eTable 20B).^42^ Cholesterol metabolism is associated with various MS disease outcomes, including disability severity and T2 lesion load.^43^ Rs28672722 is located near *HLA-DQB1,* which is associated with MS AAO at gene level (*p* = 0.007), and the SNP has been associated with allergies and cervical cancer (eTable 20C). Rs28359884, which also shows a suggestive relationship with MS severity, has been significantly associated with IL-6 levels and body-shape/waist-to-hip ratio (eTable 20D). IL-6 has been associated with MS risk and disease activity,^44,45^ and while body-shape is not causally associated with MS risk, it may be a proxy for body mass index which is an established MS risk factor and driver of poorer outcomes.^35,46^ Thus, it is possible these relationships with IL-6 and body shape might contribute to the observation relationships discussed above future work should consider comprehensive mediation analyses. Finally, the non-MHC AAO variant, rs34711, a lncRNA variant, has not been associated with other traits at the genome-wide significance threshold; however, it may be an eQTL for *MEOX2*, which is located ∼93 kb upstream. Variants in *MEOX2* have been associated with brain region volumes,^47^ and there is evidence that it may contribute to neurovascular dysfunction and neuronal loss in other neurodegenerative disorders.^48^

Gene-based tests of association revealed 46 genes were enriched for variants associated with MS AAO at *p* < 0.001 (eTable 10). Of these, 5 are HLA genes, and an additional 12 are also located in the MHC. Top hits (*p* < 1 × 10^−4^) include *HLA-C*, which contains the MS protective variant HLA-Cw*05; the BTB-Kelch–related protein *KLHL7*, which contains risk variants for Parkinson disease^49^; and *PLEK*, which has suggestive association with MS risk^50^ and significant association with spinal cord atrophy in PwMS.^51^

Several genes from the complement system were significantly associated with AAO, and they were strongly enriched in pathways derived from GS2 and GS3, for example, *PLEK* which is one of the top gene-based findings. Activation of the complement pathway enhances and expedites the innate and adaptive immune response, playing various roles in autoimmune responses, and it has been implicated in visual system degeneration in MS.^52^ Complement genes *C2*, *C4a*, and *C4b* were all enriched for MS AAO at a *p* < 0.005 (eTable 10). These genes are in the MHC Class III region, a ∼700 kb region located on chromosome 6p21.3 that separates the MHC Class I and II regions, respectively. *C2* plays a key role in activation of the classical complement response. Genetic variants in *C2* have also been associated with age-related macular degeneration in prior studies.^53^ On closer review of the gene-based association results, 8/26 (vs 1200/19136) genes involved in complement system activation^54^ were associated with AAO (*p* < 0.05), which is a 6.61-fold enrichment (95% CI 2.88–15.16; *p* = 2.8 × 10^−7^) compared with the entire genome. Of interest, no genes associated with complement system regulators/receptors^54^ were associated with AAO. Considering MS AAO is an established predictor of reaching disability milestones, this finding suggests that dysregulation in the complement system may be a shared biological mechanism. Elevated levels of *C4a* and *C4b* have been reported in the CSF of persons with relapsing-remitting MS compared with healthy controls, with elevated levels for C4a in those with active vs stable disease.^55^ *C4* has also been associated with the Expanded Disability Status Scale in MS.^55^ Considering these relationships, complement system activation may be a key mechanism that underlies the associations between AAO and MS outcomes.

The MS AAO findings were also enriched for cell signaling pathways across multiple databases, relating to the prominent MHC associations. Among the top hits for pathway enrichment, the PD-1 signaling pathway in REACTOME and the TNFα signaling through NF-kB molecular group in the Human Molecular Signatures Database are both related to programmed cell death and inflammation. Notably, the REACTOME pathway includes *HLA-DRB1* and *HLA-DQA1*; the molecular group also includes the *PLEK* gene, noted above. PD-1 and its associated ligand PD-L1 are expressed across a number of lymphocytes, as well as natural killer cells.^56^ While the exact relationship between PD-1 signaling and MS remains unclear, it seems that expression of PD-1 and its ligand play a role in neuroinflammation in MS, with varying impact on disease activity and progression.^57^ TNFα plays a complex role in the pathogenesis of MS and can exert both proinflammatory and anti-inflammatory effects in different situations.^58^ Finally, the IFN gamma signaling pathway (REACTOME) and the coagulation metabolite group (MSigDB) are among the enriched pathways for AAO. IFN gamma is secreted by a variety of activated immune cells but in MS has been shown to be a proinflammatory cytokine highly expressed in CNS lesions.^59^ In addition, recent evidence suggests that there is a connection between microglial function and chronic inflammation; the deletion of fibrinogen, a blood coagulation factor, partially reversed the neurodegenerative signatures of microglia in mouse models.^60^ Considering this and the interconnectedness of the complement system and the coagulation system, complement-associated inflammatory responses that contribute to thrombotic conditions merit further attention in MS.^61^

There was evidence from MR analyses that an earlier age of puberty contributes to an earlier onset of MS, supporting a previously published MR analysis implicating earlier puberty with MS risk.^37^ There was MR evidence that shorter telomere length conferred an earlier MS AAO. Notably, in the context of MS risk, previously published MR analyses using different genetic instrumental variables have implicated both shorter and longer telomere length with MS risk^38,39^; nonetheless, these findings indicate that select drivers of MS risk are likely to also affect AAO, as there was no evidence for associations with BMI or vitamin D levels.

This is one of the largest GWAS focused solely on MS AAO. Four independent associations, with consistent direction of effects across trials, were observed. An enrichment of the adaptive and complement immune systems was demonstrated, as well as several aspects of cell signaling related to cell death and immune activation. Future work will seek to identify additional risk loci for MS AAO by incorporating additional cohorts of PwMS. This work was limited to non-Latinx PwMS of European ancestry because of data availability, thus restricting the generalizability of our results to populations of similar ancestry. In addition, we only examined the relationships for the top MS risk and MS severity SNPs with AAO, and vice versa closer examination of variants with larger *p*-values might reveal more nuanced relationships. In addition, these clinical trials were restricted to specific MS phenotypes, with inclusion and exclusion criteria describing bounds for neurologic disability measures and age, among other factors, which are not necessarily representative of PwMS. Future analyses will seek to include diverse cohorts as they become available. Finally, MS AAO is difficult to measure. The first clinical symptom of MS may not directly correspond with age at the development of MS lesions; most CNS lesions are asymptomatic. This could introduce variability into our study results.

Two novel genetic loci associated with MS AAO were identified in this meta-analysis of 6 clinical trials of PwMS. There was an enrichment in adaptive and complement immunity, as well as various cell signaling pathways related to cell death and immune response. There was also evidence supporting a link with age at puberty and telomere length. We note that these AAO variants were also associated with MS risk, which is largely driven by dysregulated immune response, with a possible connection for one variant with MS severity, which is largely driven by processes related to CNS resilience. Overall, the findings suggest that AAO in MS is multifactorial, and specific immunologic mechanisms and risk factors that contribute to MS risk also influence the onset of symptoms.

## Appendix Authors

**Table:**

Name	Location	Contribution
Elina Misicka, PhD	Department of Population and Quantitative Health Sciences, Case Western Reserve University, Cleveland, OH	Drafting/revision of the manuscript for content, including medical writing for content; major role in the acquisition of data; study concept or design; analysis or interpretation of data
Yunfeng Huang, PhD MPH	Biogen Inc., Cambridge, MA	Drafting/revision of the manuscript for content, including medical writing for content; major role in the acquisition of data; study concept or design; analysis or interpretation of data
Stephanie Loomis, PhD, MPH	Biogen Inc., Cambridge, MA	Major role in the acquisition of data; analysis or interpretation of data
Nilanjana Sadhu, PhD	Biogen Inc., Cambridge, MA	Major role in the acquisition of data; analysis or interpretation of data
Elizabeth Fisher, PhD	Biogen Inc., Cambridge, MA	Major role in the acquisition of data; analysis or interpretation of data
Arie Gafson, MBBS PhD	Biogen Inc., Cambridge, MA	Major role in the acquisition of data; analysis or interpretation of data
Heiko Runz, MD	Biogen Inc., Cambridge, MA	Major role in the acquisition of data; analysis or interpretation of data
Ellen Tsai, PhD	Biogen Inc., Cambridge, MA	Major role in the acquisition of data; analysis or interpretation of data
Xiaoming Jia, MD	Human Genetics and Bioinformatics, Genentech, San Francisco, CA	Major role in the acquisition of data; analysis or interpretation of data
Ann Herman, PhD	Human Genetics and Bioinformatics, Genentech, San Francisco, CA	Major role in the acquisition of data; analysis or interpretation of data
Paola G. Bronson, PhD	Biogen Inc., Cambridge, MA	Drafting/revision of the manuscript for content, including medical writing for content; major role in the acquisition of data; study concept or design; analysis or interpretation of data
Tushar Bhangale, PhD	Human Genetics and Bioinformatics, Genentech, San Francisco, CA	Drafting/revision of the manuscript for content, including medical writing for content; major role in the acquisition of data; study concept or design; analysis or interpretation of data
Farren B. Briggs, PhD, ScM	Department of Public Health Sciences, University of Miami, FL	Drafting/revision of the manuscript for content, including medical writing for content; major role in the acquisition of data; study concept or design; analysis or interpretation of data

## Glossary

AAO: age at onset
BMI: body mass index
GWAS: genome-wide association study
IMSGC: International MS Genetics Consortium
MHC: major histocompatibility complex
MS: multiple sclerosis
PPMS: primary-progressive MS
PwMS: persons with MS
RRMS: relapsing-remitting MS
SNPs: single nucleotide polymorphisms
SPMS: secondary-progressive MS
